# Is a High Tone Pointy? Speakers of Different Languages Match Mandarin Chinese Tones to Visual Shapes Differently

**DOI:** 10.3389/fpsyg.2017.02139

**Published:** 2017-12-07

**Authors:** Nan Shang, Suzy J. Styles

**Affiliations:** Psychology, School of Social Sciences, Nanyang Technological University, Singapore, Singapore

**Keywords:** cross-modal correspondences, sound symbolism, Mandarin Chinese tones, language-specific perception, bouba/kiki test

## Abstract

Studies investigating cross-modal correspondences between auditory pitch and visual shapes have shown children and adults consistently match high pitch to pointy shapes and low pitch to curvy shapes, yet no studies have investigated linguistic-uses of pitch. In the present study, we used a bouba/kiki style task to investigate the sound/shape mappings for Tones of Mandarin Chinese, for three groups of participants with different language backgrounds. We recorded the vowels [i] and [u] articulated in each of the four tones of Mandarin Chinese. In Study 1 a single auditory stimulus was presented with two images (one curvy, one spiky). In Study 2 a single image was presented with two auditory stimuli differing only in tone. Participants were asked to select the best match in an online ‘Quiz.’ Across both studies, we replicated the previously observed ‘*u*-curvy, *i*-pointy’ sound/shape cross-modal correspondence in all groups. However, Tones were mapped differently by people with different language backgrounds: speakers of Mandarin Chinese classified as Chinese-dominant systematically matched Tone 1 (high, steady) to the curvy shape and Tone 4 (falling) to the pointy shape, while English speakers with no knowledge of Chinese preferred to match Tone 1 (high, steady) to the pointy shape and Tone 3 (low, dipping) to the curvy shape. These effects were observed most clearly in Study 2 where tone-pairs were contrasted explicitly. These findings are in line with the dominant patterns of linguistic pitch perception for speakers of these languages (pitch-change, and pitch height, respectively). Chinese English balanced bilinguals showed a bivalent pattern, swapping between the Chinese pitch-change pattern and the English pitch-height pattern depending on the task. These findings show for that the supposedly universal pattern of mapping linguistic sounds to shape is modulated by the sensory properties of a speaker’s language system, and that people with high functioning in more than one language can dynamically shift between patterns.

## Introduction

For almost 90 years, it has been recognized that people from a variety of backgrounds tend to make the same choices about which nonsense words ‘should’ have which meanings, a trait that seems to be more-or-less universal. For example, English speakers showed high levels of agreement in judging a word form like *mal* to be a better match for a larger object than *mil* (Sapir, 1929). Similarly, most people preferred to match curvy line drawings with the nonsense word *baluba* (*maluma* in the 1947 version) and angular line drawings with *takete* (Köhler, 1929/1947/1970). The same sound-shape mapping pattern has also been documented by Ramachandran and Hubbard (2001) who found that the majority of participants (95%∼98%) matched a curvy shape with *bouba* and an angular shape with *kiki*—this effect has since become known as the *bouba-kiki* effect. The *bouba-kiki* paradigm has been replicated cross-linguistically and cross-culturally, for example, with Swahili-speaking school children living in an isolated peninsula in Africa (Davis, 1961), Czech-speaking adults (Tarte, 1974), Tamil speakers in India (Ramachandran and Hubbard, 2001) and Otjiherero-speaking Himba living in Northern Namibia (Bremner et al., 2013). The *bouba-kiki* effect has also been found in pre-reading toddlers (Maurer et al., 2006), in pre-vocabulary-spurt 11-month-olds (Imai et al., 2008; Kantartzis et al., 2011; Imai and Kita, 2014), and in pre-linguistic 4-month-olds (Ozturk et al., 2013). These experiments suggest that the effect has its origins prior to the acquisition of language, and is therefore not dependent on language learning.

Some researchers have suggested that these effects are related to the generalized sensory confusion in newborn children, described as a kind of ‘neonatal synaesthesia’ preceding clear sensory differentiation (Maurer, 1993), which may give rise to a kind of ‘weak synesthesia’ in adulthood, from latent sensory ‘cross-wiring’ (Ramachandran and Hubbard, 2001) that remains after developmental changes in connectivity and function (Maurer and Mondloch, 2005). Others view linguistic sound symbolism as an offshoot of generalized cross-modal processing, acquired through experience with the structural regularities of sensory information, as derived from the physical environment (Spence, 2011; Spence and Deroy, 2013). To give an example of environmental regularities, small resonating bodies produce high-pitched sounds, whereas larger bodies are capable of lower-pitched sounds (e.g., trumpet/tuba; mosquito/elephant). These perspectives converge on the idea that the cross-modal perception underlying the bouba/kiki effect is universal (although notable exceptions include Rogers and Ross, 1975 and Styles and Gawne, 2017).

Outside the domain of language, crossmodal congruences between auditory pitch and other sensory modalities have been widely investigated (see Spence and Deroy, 2013 for a review). Early empirical evidence of pitch-related cross-modal correspondences comes from Marks’ laboratory experiments. For example, when given a set of colors varying in lightness and a set of notes varying in pitch, participants consistently paired the higher pitch with the lighter color (Marks, 1978). He also found cross-modal correspondences between pitch and direction as well as pitch and sharpness (Marks, 1974, 1987). In one experiment, Marks (1987) asked participants to match two auditory stimuli (one 220-Hz saw-tooth wave and one 360-Hz saw-tooth wave) to two visual forms (one U-shape and one V-shaped, respectively) and found that the high pitched sound was matched to the angular shape and the low pitched sound was matched to the round shape. O’Boyle and Tarte (1980) also found that when asked to adjust pure tone frequencies to best fit visual stimuli, participants more often assigned lower frequencies to round figures than to angular figures. Auditory pitch is also matched with visual size, for example, Gallace and Spence (2006) asked participants to judge relative visual sizes of objects accompanied by task-irrelevant sounds, and found faster responses to congruent trials (e.g., a big disk with a low pitch sound) than to incongruent ones (e.g., a big disk with a high pitch sound). Pitch-related cross-modal correspondences have also been confirmed in young children (e.g., pitch and size; pitch and brightness in Mondloch and Maurer, 2004) and infants (e.g., pitch and visuospatial height; pitch and visual sharpness in Walker et al., 2010); pitch and size in Fernández-Prieto et al. (2015). Ludwig et al. (2011) even observed the high-high mapping pattern between pitch and luminance in chimpanzees, as has been observed in humans, suggesting such mappings were present in our common ancestors. Taken together, previous studies have shown cross-modal correspondences between pitch and vision, including visual angularity. All of these experiments have been conducted using pitch stimuli with no linguistic content. Linguistic uses of pitch, however, have not been systematically investigated in the cross-modal correspondence literature.

**FIGURE 1:**
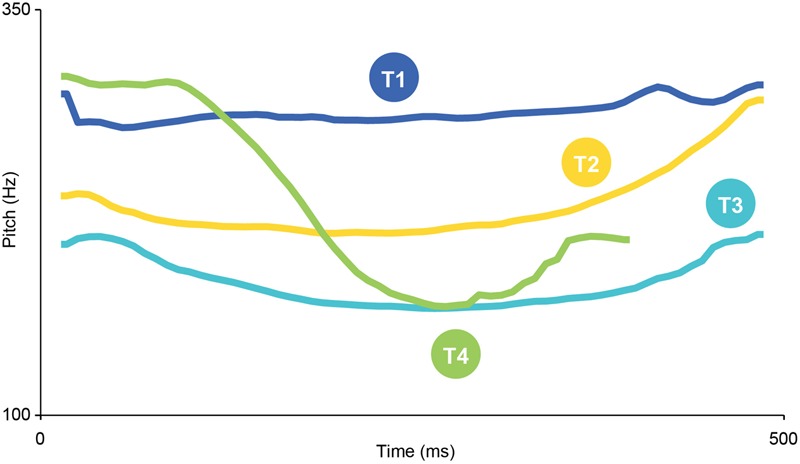
Example pitch contours of the four Mandarin tones, as measured in PRAAT (Boersma, 2001).

Lexical tones refer to syllable-level variations in the temporal contour of the fundamental frequency (F0, or ‘pitch’) and serve to draw contrasts between word meanings (Gandour, 1978; Jongman et al., 2006; Singh and Fu, 2016). Lexical tones exist in about 70% of the world’s languages (Yip, 2002) and over half the world’s population speak a tone language (Fromkin, 1978). However, cross-modal correspondences for speakers of tone languages have rarely been investigated. Mandarin Chinese is a tone language with four lexical tones. In terms of pitch, Tone 1 is high and steady, Tone 2 mid-rising, Tone 3 falling-rising and Tone 4 high-falling (Chao, 1948), as can be seen in **Figure 1**. Tone information is vital for meaning discrimination in Mandarin Chinese. For example, when a syllable like ‘yan’ is produced in the four tones, each word has a distinct meaning: 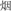 (*yan1*, ‘smoke’); 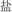 (*yan2*, ‘salt’); 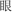 (*yan3*, ‘eye’); 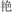 (*yan4*, ‘colorful’).

Only a few studies have investigated sound symbolism in Mandarin Chinese or with Mandarin-speaking participants. Sapir’s early work on vowel correspondences ([a]-big, [i]-small) included Chinese-speaking participants, who performed the same way as their Western peers (Sapir, 1929). Similarly, Huang et al. (1969) and Chan (1996) have reported the ‘[a]-big, [i]-small’ sound-size mapping pattern in lexical items of Chinese. These findings suggest that Chinese speakers share general sound symbolic mapping patterns, when it comes to sounds that are highly prevalent and contrastive, like the ‘corner vowels’ [i], [a], and [u], which are common to the majority of spoken languages (Styles and Gawne, 2017). A small number of additional studies have suggested that English speakers share sufficient sound symbolic mapping patterns with Chinese speakers that they can guess the correct meanings of Chinese word pairs (e.g., antonym pairs) when words are written phonetically (Brackbill and Little, 1957; Weiss, 1963; Klank et al., 1971) or spoken (Brown et al., 1955; Brown and Nuttall, 1959), at levels better than predicted by chance. However, in a slightly different method, LaPolla (1994) asked native English speakers with no knowledge of Chinese to pick the correct meaning from of a pair of Chinese antonyms, in two versions of the same task – one which included the original words with tones articulated correctly, and a second version where the tones were swapped between antonyms in each pair. LaPolla’s curious finding was that the English speakers performed *better* when tones in the Chinese antonym pairs were swapped. In another experiment from the same paper (LaPolla, 1994), he found that Mandarin speakers showed chance performance for sound-size mapping when listening to Cantonese minimal pairs differing only in tones. It is important to note here that Mandarin Chinese and Cantonese have radically different tone systems. Both of these findings undermine the supposed universality of sound symbolism when it comes to people who do/don’t speak a tone language, or who speak languages with different tone systems. To date, no satisfactory explanation has been proposed as to how or why these tone-based anomalies exist. Given the fact that so many people in the world speak tone languages and sound symbolism has not been investigated systematically using tone languages, the present study explores cross-modal correspondences between Mandarin tones and visual shapes in two highly systematic investigations of bouba/kiki-type sound-shape matching.

In previous research investigating which sounds match with which shapes, Nielsen and Rendall (2011, 2013) found that sonorant consonants (/m/, /n/, or /l/) and rounded vowels (“oo,” “oh” or “ah”) were matched to curved images while voiceless plosive consonants (/t/, /k/ or /p/) and non-rounded vowels (“ee,” “ay” or “uh”) were matched to jagged images. Similarly, D’Onofrio (2014) found that non-words containing voiced consonants (/b/, /d/ or /g/), labial consonants (/b/ or /p/) and back and/or rounded vowels (/u/ or /a/) were matched with round shapes more than their respective counterparts (/t/, /k/; /i/, /e/). Recently, Fort et al. (2015) also replicated the ‘[o], [u]-round, [i], [e]-spiky’ audiovisual mapping pattern. Taken together, these studies suggest that sonorants, voiced stops and back rounded vowels typically match with curvy shapes, voiceless stops and high-front unrounded vowels typically match with pointy shapes (c.f. Köhler, 1929/1947/1970; Davis, 1961; Ramachandran and Hubbard, 2001; Maurer et al., 2006; Nielsen and Rendall, 2011, 2013; D’Onofrio, 2014; Ozturk et al., 2013; Fort et al., 2015). Hence, all previous studies agree that the high front non-rounded vowel [i] (the ‘ee’ vowel in ‘feet’) and the high back rounded vowel [u] (the ‘oo’ vowel in ‘shoe’) represent a highly salient pointy-curvy contrast as [i] and [u] representing two extremes of vowel space for the majority of documented languages (cf. Styles and Gawne, 2017). Notably, most of the stimuli used in earlier studies differ in multiple phonetic features, making it hard to tease apart the detailed source of the effects. For example, in Köhler’s earliest evidence, *maluma* differs from *takete* in vowel roundedness, vowel backness, sonority of consonants, continuity of consonants, voicing of consonants as well as place of articulation of consonants. Most of these features are also contrasted in *bouba* and *kiki* (Ramachandran and Hubbard, 2001), as well as the nonsense word-pairs used in Maurer et al. (2006). Since the focus of the current study is the sound-symbolic congruence for tones, we elected to test the smallest acoustic element that can carry a tone – a vowel produced in isolation. This decision allows precise control of the non-tone elements of the speech, and removes possible confounds between vowels and consonants. Furthermore, tones are documented to be more easily identified when presented in isolation (i.e., monosyllables, Broselow et al., 1987). The current study therefore investigates sound symbolism for the single vowels [i] and [u] articulated in the four Mandarin tones.

The two authors had different predictions for what would happen. Given the extensive literature on cross-modal correspondences between pitch and visual shapes (O’Boyle and Tarte, 1980; Marks, 1987; Walker et al., 2010; Parise and Spence, 2012), the second author, a native English speaker who does not speak Chinese, predicted that the high-pitched Tone 1 would be matched with pointy shapes, while the low-pitched Tone 3 would be matched with smooth curvy shapes. By contrast, the first author, based on her experience as a native speaker of Mandarin Chinese, predicted a different pattern: the smooth, steady Tone 1 would be mapped with curvy shapes, while the dynamically changing Tone 4 would be mapped with visually dynamic pointy shapes. Because of our radically different expectations (Tone 1, pointy; Tone 1, curvy), we chose to compare speakers with different experience of the Mandarin tone system. Both authors expected to replicate the well documented [u]-curvy, [i]-pointy vowel-shape pattern (e.g., D’Onofrio, 2014). To date, the present study is the first to investigate lexical tones in systematically controlled sound symbolic selection paradigm (a modified bouba/kiki task).

## Study 1: Two Shapes with One Sound

### Materials and Methods

Participants were invited to take part in an online quiz using social media. The quiz consisted of eight audiovisual questions, followed by demographic questions. The experimental procedure was approved by the IRB of Nanyang Technological University.

#### Participants

One hundred and fourteen volunteer participants (64 females), aged 18–57 years, took part in the present study, conducted using Qualtrics online survey software. Participants were over 18 years of age and completed the Information and Consent page online. We designed the study for three groups: a Chinese dominant bilingual group (C); a Chinese-English balanced bilingual group (C/E) and a group of English speakers with no Chinese (E). No fixed limits were set for group size in the online data collection. The study was closed on a predetermined day shortly after 100 participants were recorded, and before analysis was conducted. According to the predetermined grouping criteria, 14 participants did not fall into one of these groups, and were excluded. Therefore, there were 100 valid participants in the present study (C: 45; C/E: 30; E: 25).

#### Grouping Criteria

A single question asked participants if they are bilinguals of English-and-Chinese. Participants were also asked to rate their proficiency in each of their languages and dialects on a five-point scale where one represents highest competence and five represents lowest, and zero represents that the participant has no knowledge of that language. For further details of the language questions, see the Supplementary Materials for this article. Participants were also asked about where they live now and their residence history in different life stages. Participants were allocated to the C group (Chinese dominant) if they identified as English-and-Chinese bilinguals, their Chinese was self-reported at the highest level, their English was self-reported as lower, and they completed all schooling up to undergraduate level in Mainland China. Participants were allocated to the C/E group (Chinese–English balanced bilinguals) if they identified as English-and-Chinese bilinguals, their Chinese and English proficiency differed by no more than 1 point on the scale, and they completed all schooling up to undergraduate level in Singapore. Participants were allocated into the E group (English speakers with no tone language experience), if they had no knowledge of Chinese and their English was self-reported to be native or near-native level. Group E reported their schooling in a variety of countries (e.g., Singapore, United Kingdom, United States, Australia, Germany, etc.).

#### Stimuli

**FIGURE 2:**
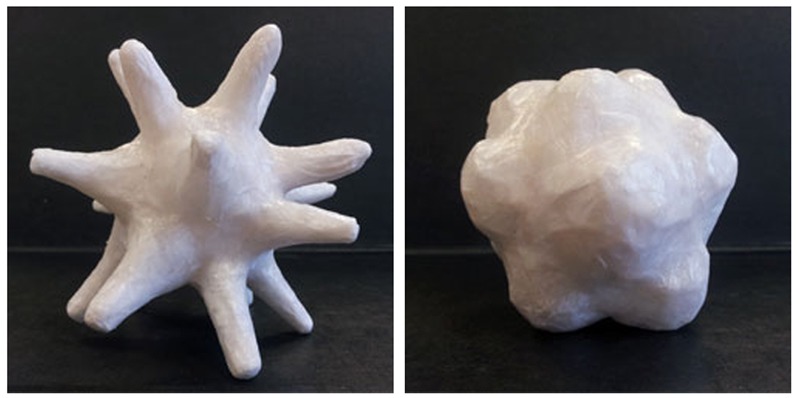
Visual stimuli: Two hand-made novel three-dimensional objects capturing salient bouba/kiki contrast dimensions. For original photographs, see the Open Science Framework Repository for this Project (https://osf.io/364fm).

The visual stimuli were two ivory three-dimensional novel objects (one curvy; one pointy), photographed against a black background (see **Figure 2**). The hand-made objects captured salient bouba/kiki differences. The 3D forms were designed to be more visually interesting than more-familiar 2D line drawings, so that participants could maintain visual interest over multiple test trials.

The auditory stimuli were the high front non-rounded vowel [i] (the ‘ee’ vowel in ‘feet’) and the high back rounded vowel [u] (the ‘oo’ vowel in ‘shoe’) articulated in the four tones of Mandarin Chinese by a female native speaker. For recording, the auditory stimuli were produced a minimum of three times each as isolated monosyllables, with the syllables arranged in a number of different orders to ensure variation across the recorded set of stimuli. The auditory stimuli were recorded in a sound-proof recording lab using a Shure SN81 microphone and Acoustica recording software, at a 44.1 kHz sampling rate with 16 bit encoding. Audio were edited and trimmed using GoldWave software.

#### Auditory Stimulus Selection and Validation

To ensure that the audio tokens were sufficiently standard for use in the test, we asked seven bilingual speakers of Mandarin Chinese and English to evaluate the typicality of each recording as an exemplar of the tone produced on the vowel in question. We played each sound, and asked what vowel it was, what tone it was, and asked people to rate the typicality of each sound, on a scale from one to seven. Seven represented the most typical and one represented the least typical. People could also mark zero, if they thought it did not sound like the category at all. All seven raters agreed on the identity of the vowels and the tones.

**FIGURE 3:**
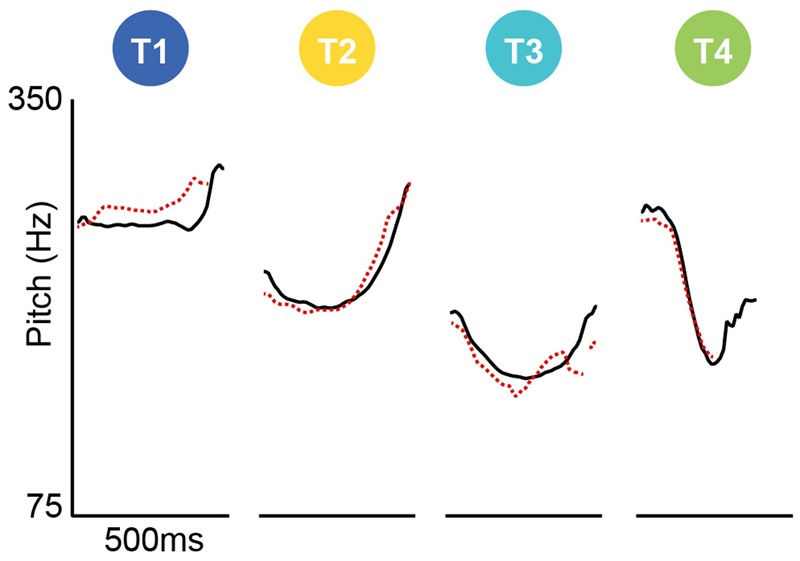
Pitch tracks showing pitch (Hz) as it evolves over the 500 ms of the eight auditory stimuli: Vowels [i] (red lines) and [u] (black lines) articulated in each of the lexical tones of Mandarin Chinese (T1, T2, T3, T4). For original audio files, see the Open Science Framework Repository for this Project (https://osf.io/364fm).

The highest typicality token of each stimulus was selected for use in the studies reported here. All stimuli were rated as very typical, with median scores of 5 or 6. Each of the eight sound files was 500 ms long. **Figure 3** shows the pitch tracks of the eight auditory stimuli as measured in PRAAT (Boersma, 2001), where it is clear that the [i] and [u] track within each tone are more similar to each other than are the pitch tracks between tones. That is to say, each tone was clearly differentiated from the others, and its contour was consistent across the two stimuli. As can be seen in **Table 1** and **Figure 2**, mean pitch differs most between Tone 1 and Tone 3, and pitch variance differs most between Tone 1 and Tone 4.

**Table 1:** Pitch measurements for the eight auditory stimuli used in this study.

Stimulus	Pitch onset (Hz)	Pitch offset (Hz)	Mean pitch (Hz)	Pitch SD (Hz)	Variance (Hz)	Δ F0 (Max–Min)
u1	281.0	301.0	286.4	6.2	53.0	24.3
u2	236.4	294.2	229.5	21.7	107.1	81.9
u3	209.3	213.3	166.1	15.4	92.5	47.4
u4	308.1	208.8	220.6	53.7	188.4	140.9
i1	279.7	307.7	296.9	7.4	44.9	28.4
i2	223.1	290.4	229.3	24.9	96.1	81.1
i3	202.2	189.4	181.9	13.2	83.6	117.6
i4	280.5	212.3	224.5	39.9	153.3	106.9

#### Experimental Platform

Following a pilot study reported elsewhere, the experiment was presented using Qualtrics online survey software. Participants were instructed to run the experiment using laptops or computers, since the platform did not guarantee stable audio on mobile devices at the time. Participants were also instructed to use headphones and to do the online quiz in a quiet environment.

#### Procedure

Participants were presented with a single audio file and a pair of pictures, and were asked “Which of these two shapes goes better with this sound.” In the online procedure, after obtaining consent and adjusting audio volume, participants were presented with eight experimental pages followed by the demographic and language background questions. In each experimental page, participants were presented with two 250 px × 250 px pictures side-by-side along with a button which triggered the audio file to play. In each question, participants could listen to the auditory stimulus as many times as they liked, and they were asked to decide which of the two visual stimuli was a better match for the sound. Each of the eight questions was presented on a separate page, and each page was unreturnable. The location of the two pictures (right, left) was randomized, as was the presentation sequence of test questions. The whole procedure took around 7 min. The materials and precise instructions for the task can be found in the Open Science Framework repository for this project^1^.

#### Predictions

According to previous European-language sound-shape matching tasks (e.g., D’Onofrio, 2014), we expected all participants would show an [i]-pointy, [u]-curvy preference, a ‘vowel effect.’ If speakers with different language backgrounds differ in their perceptual mapping preferences (as did the two authors), then different groups would show different mapping patterns. In particular, if crossmodal perception of tones and shapes is guided by *pitch change*, since Chinese speakers are sensitive to pitch change, we expected that the Chinese speakers would show different responses to Tone 1 and Tone 4, as Tone 1 has the least pitch change while Tone 4 has the most pitch change. Hence, by paying attention to pitch change, Chinese speakers would match steady Tone 1 with the curvy shape and dynamic Tone 4 with the pointy shape. If on the other hand, *pitch height* is the major driver of this kind of crossmodal correspondence, as English speakers’ tone processing mainly focuses on pitch height (Gandour, 1983), the English speakers may show different responses to Tone 1 (high-pitched) and Tone 3 (low-pitched). Hence, by paying attention to pitch height, English speakers would match high Tone 1 with the pointy shape and low Tone 3 with the curvy shape.

#### Analytical Approach

To investigate the influence of vowel ([i], [u]), tone (T1, T2, T3, T4), and language group (C, C/E, E) and the interactions among them on shape choice, we ran a fully factorial generalized linear mixed model (GLMM) test, with participant as the only random factor. Since the outcomes are dichotomous (curvy or pointy), we used Binary logistic regression that couples a binomial probability distribution with the logit link function (which is the canonical link function for the binomial distribution) (Heck et al., 2012). Between group effects and interactions were followed up with pairwise group comparisons in GLMM. Tone effects were followed up with Related-Samples McNemar tests to compare pairs of tones. The full statistical reports for each GLMM test can be found in the Supplementary Materials, along with the results of pair-wise comparisons, and Related-Samples McNemar tests, with summary data presented here in the Results section.

### Results

**FIGURE 4:**
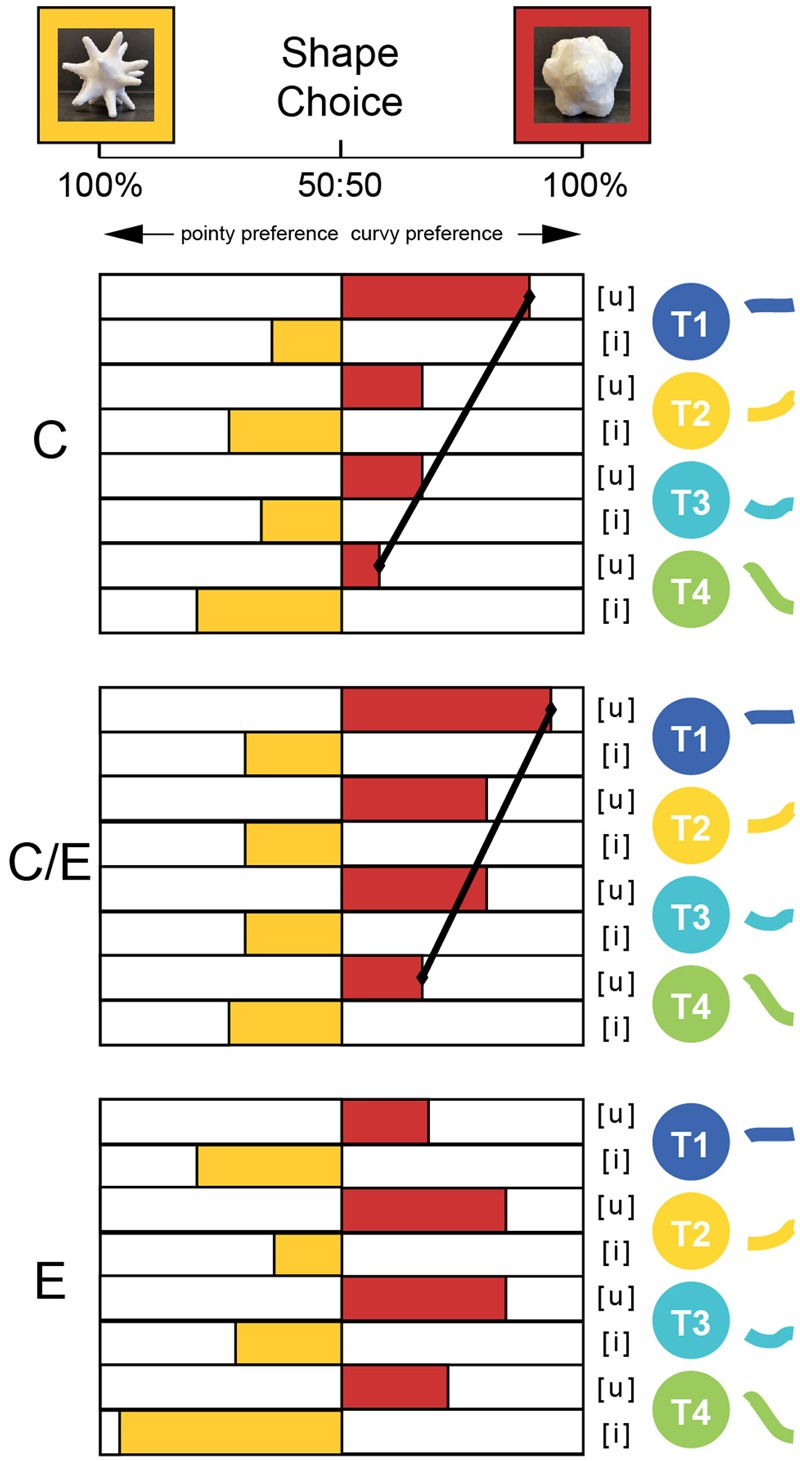
Shape choice for the three groups for Study 1, where a single vowel was presented with two visual shapes. Choices shown separately for each vowel ([i], [u]) articulated in each of the four Chinese Tones (T1, T2, T3, T4). Groups of participants were C: Chinese-dominant educated in China; C/E: Chinese–English balanced bilinguals educated in Singapore: E: English-speakers with no knowledge of Chinese. Black angled lines indicate significant within-group tone contrasts for tones produced on the same vowel.

**Figure 4** shows the percentage of participants who selected the pointy or the curvy shape for each of the eight sounds, with language groups shown separately, following the graphical logic of Fort et al. (2015).

According to the GLMM test, the main effect of vowel [*F*(1,776) = 140.11, *p* < 0.001] and the main effect of tone [*F*(3,776) = 4.54, *p* = 0.004] were significant. The log odds of choosing the curvy shape were higher for the [u] vowel (β = 4.123, *p* < 0.001) than for the [i] vowel, holding the other effects constant. Overall, all participants, regardless of language background, made significantly more curvy choices for [u] than for [i]. This preference for the ‘[i]-pointy, [u]-curvy’ matching pattern is in line with the existing literature.

In unpacking the tone effect, holding all other effects constant, the log odds of choosing the curvy shape were higher for Tone 1 than Tone 4 (β = 3.178, *p* = 0.002), Tone 2 than Tone 4 (β = 2.603, *p* = 0.02) and Tone 3 than Tone 4 (β = 2.234, *p* = 0.049). Overall, there was a T4 pointy effect with T1 the most curvy and T2 and T3 in between. The interaction between tone and language group did not achieve significance [*F*(3,776) = 1.867, *p* = 0.084]. However, since visual inspection of **Figure 3** suggests somewhat different response patterns for the [i] and [u] vowels, further analyses were conducted on responses to [i] and [u] stimuli separately.

For the [i] stimuli, there was a significant main effect of tone [*F*(3,388) = 4.05, *p* = 0.007] that did not interact with language groups. The log odds of choosing the pointy shape for Tone 4 were higher than for other tones (T1: β = 1.962, *p* = 0.031; T2: β = 2.912, *p* = 0.001; T3: β = 2.476, *p* = 0.007), holding the other effects constant.

For the [u] stimuli, there was a significant main effect of tone [*F*(3,388) = 4.81, *p* = 0.003] and a significant interaction between tone and language group [*F*(6,388) = 2.82, *p* = 0.011]. The log odds of choosing the curvy shape for Tone 1 were higher in the Chinese-dominant group (β = 2.1, *p* = 0.006) and the Chinese–English balanced group (β = 2.436, *p* = 0.008) than in the English group, holding the other effects constant. Overall, the two Chinese groups showed a similar response pattern (i.e., T1 curvy, T4 pointy, T2 and T3 in between), whereas the English group did not (i.e., T1 and T4, in particular T1, was pointier than T2 and T3 for the E group). To unpack this interaction, we ran pair-wise comparisons between language groups.

In the comparison between C group and E group, the difference in tone was also significant [*F*(3,272) = 3.78, *p* = 0.011]. Holding the other effects constant, the log odds of choosing the curvy shape for Tone 1 were higher in the Chinese-dominant group (β = 2.04, *p* = 0.01) than in the English group. The pairwise comparisons demonstrated that the C group made significantly more curvy choices for [u] stimuli articulated in Tone 1 than in Tone 4 (C/E: *p* = 0.003), with T2 and T3 falling between T1 and T4. The English group showed no significant results for any pairwise comparison between tones for the [u] vowel. However, visual inspection of the graph suggested that English speakers may be treating Tone 1 and 4 differently from Tone 2 and 3. These pairs are of interest as Tone 1 and Tone 4 share a high pitch onset, while Tone 2 and Tone 3 share a low pitch onset, and English-speakers’ perceptual sensitivity for high/low contrasts may be strongest at the onset of Mandarin Chinese tones, where the pitch information is typically loudest. In a follow-up exploratory analysis, the comparison between the pooled T1, T4 and the pooled T2, T3 was significant (*p* = 0.041, uncorrected). Hence, the English-speaking participants did not show the Chinese-tone mapping pattern, but tended to make somewhat more pointy choices for T1 and T4 than for T2 and T3.

In the comparison between the CE and the E group, the tone effect differed significantly [*F*(3,212) = 3.01, *p* = 0.031]. The log odds of choosing the curvy shape for Tone 1 were higher in the Chinese–English balanced group (β = 2.414, *p* = 0.01) than in the English group, holding the other effects constant. We also conducted pairwise comparisons (Related-Samples McNemar Tests) between the tones for each group. The Related-Samples McNemar tests showed that, similar to the C group, the CE group also showed the significant ‘T1-curvy, T4-pointy’ response pattern (C: *p* = 0.007), with T2 and T3 between them.

When comparing the two Chinese groups, there was a significant main effect of tone [*F*(3,292) = 12.28, *p* < 0.001], but this effect did not differ between the two Chinese groups. The log odds of choosing the curvy shape for Tone 1 were higher (β = 2.432, *p* < 0.001) than for Tone 4, holding the other effects constant. Hence, both Chinese-speaking groups show the same response pattern where T1 was the curviest, T4 the pointiest, and T2 and T3 between them.

Taking the pairwise comparisons together, it is clear that Chinese speakers in both groups showed a ‘T1-curvy, T4-pointy’ pattern, and both groups differed from English speakers who showed a ‘T1/T4 pointy, T2/T3 curvy’ pattern for [u].

### Discussion

Consistent with the results of previous studies (Köhler, 1929/1947/1970; Ramachandran and Hubbard, 2001; D’Onofrio, 2014), the ‘*u*-curvy, *i*-pointy’ cross-modal correspondence was replicated in our online 2AFC survey, across participants with different language backgrounds, further supporting the consistency of the cross-modal correspondences for these high-prevalence vowels.

In addition to the vowel effect, Chinese speakers (both C Group and C/E Group) made more curvy choices for [u] stimuli articulated in Tone 1 than in Tone 4, which is consistent with the previous literature on Chinese speakers’ sensitivity to pitch change (Jongman et al., 2006; Singh et al., 2014), and demonstrates for the first time that this perceptual sensitivity is also evident in crossmodal perception using two large samples of homogeneous Chinese speakers (one Chinese dominant with predominately Mandarin Chinese language experience, the other bilingually educated Singaporeans with self-reported balance in their language skills in Chinese and English). This finding also replicated the observations of the pilot study (reported elsewhere), suggesting a robust, replicable effect.

**FIGURE 5:**
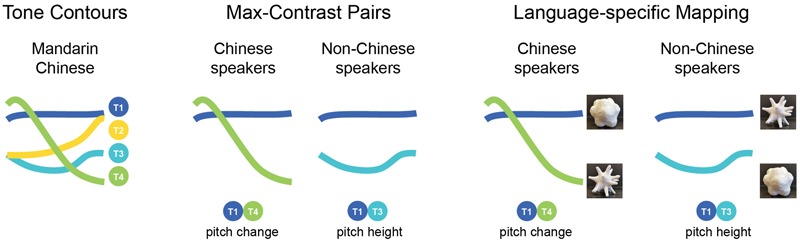
Schematic diagram illustrating the Tone Contours for the four tones of Mandarin Chinese; The Maximum Contrast Tone-pairs for each group of speakers (as established in the previous literature), and the Language-specific Mapping patterns observed in Study 1, for conceptual replication in Study 2.

By contrast, English-speaking participants made fewer curvy choices for Tone 1 and Tone 4 compared to for Tone 2 and Tone 3. This pattern was only significant for the [u] vowel, when analyzed in isolation, and the interaction between vowel, tone and group was not significant, meaning that this exploratory finding should be treated with caution until further replicated (see Experiment 2). In unpacking the direction of this trend, it should be noted that Tone 1 and Tone 4 both have high pitch onsets compared to Tone 2 and Tone 3. In other words, instead of pitch change, the English speakers tend to pay more attention to pitch height. This finding is in line with the previous literature on Western high/low pitch perception in lexical tones (Gandour, 1983) and in non-linguistic crossmodal pitch processing (O’Boyle and Tarte, 1980; Marks, 1987; Walker et al., 2010). In **Figure 5**, we summarize these patterns graphically.

In the present study, each stimulus carried vowel identity information (tongue height/backness and lip rounding) as well as tone identity information. As observed here, all groups of participants showed a strong ‘[i]-pointy, [u]-curvy’ matching pattern, with tone modulating responses less strongly. The tone effect was observed for [u] but was not observed for [i]. Since vowel identity is such a strong predictor of shape choice, it may have overshadowed observation of a subtler tone effect. Furthermore, since non-tone language speakers are known for their inability to hold representations of tone information in short term memory, hearing each tone in a separate trial may have ‘washed out’ perceptual effects that may be evident if the tone categories are contrasted more explicitly. For this reason, we developed a second study designed to make the perceptual differences between pitch contours more salient even for non-tone language participants, by presenting two syllables varying only in tone (a tone-minimal pair) along with a single shape.

## Study 2: Two Sounds with One Shape

### Materials and Methods

The quiz consisted of 12 audiovisual questions, followed by demographic questions, implemented as in Study 1. The experimental procedure was approved by the IRB of Nanyang Technological University.

#### Participants

In total 104 participants (68 females), aged 18–57 years participated in this online quiz. Allocation of participants to groups was conducted as in the previous study, resulting in 31 participants in the Chinese dominant bilingual group (C); 24 participants in the Chinese-English balanced bilingual group (C/E) and 49 participants in the English speakers with no Chinese group (E).

#### Stimuli

The stimuli from Study 1 were recombined into a task where one visual shape was presented with two auditory stimuli differing only in tone (a tone minimal pair), i.e., vowels within a pair were always the same, and minimal pairs were created for each vowel. The full procedure included all tone minimal pairs. The critical conditions of interest are the T1–T3 pair and the T1–T4 pair, which are maximally contrastive for pitch height, and pitch change, respectively. Other tone pairs were included as fillers.

#### Procedure

In each question, participants were presented with one picture of a shape (curvy or pointy) and a button which triggered an audio file including a minimal pair of sounds separated by a brief silence of 500 ms duration. Participants were asked which of the two sounds (the first or the second) went better with the shape. The remaining procedures were identical to that of Study 1.

#### Predictions

Based on the results of Study 1, we expected that the Chinese groups would show a pitch-change-driven mapping pattern by matching T1 with the curvy shape, T4 with the pointy shape. On the other hand, the English group would show a pitch-height-driven mapping pattern by matching T1 with the pointy shape, T3 with the curvy shape as illustrated in **Figure 5**. Note that this means different groups would match T1 to opposite shapes. We further predicted that experimental control of vowel would result in the tone effects being equal for the two vowels.

#### Analytical Approach

The same approach was taken as in Study 1, and full statistical reports for Study 2 can be found in the Supplementary Materials.

### Results

Given the suggestions from Study 1 that pitch change is important for Chinese speakers and pitch height is important for English speakers in their tone-shape mappings, our interest here is whether pitch-change or pitch-height is the main driver of tone-shape mappings for different groups of participants, when vowel identity is controlled.

#### T1/T4 – The Pitch-Change Pair

**FIGURE 6:**
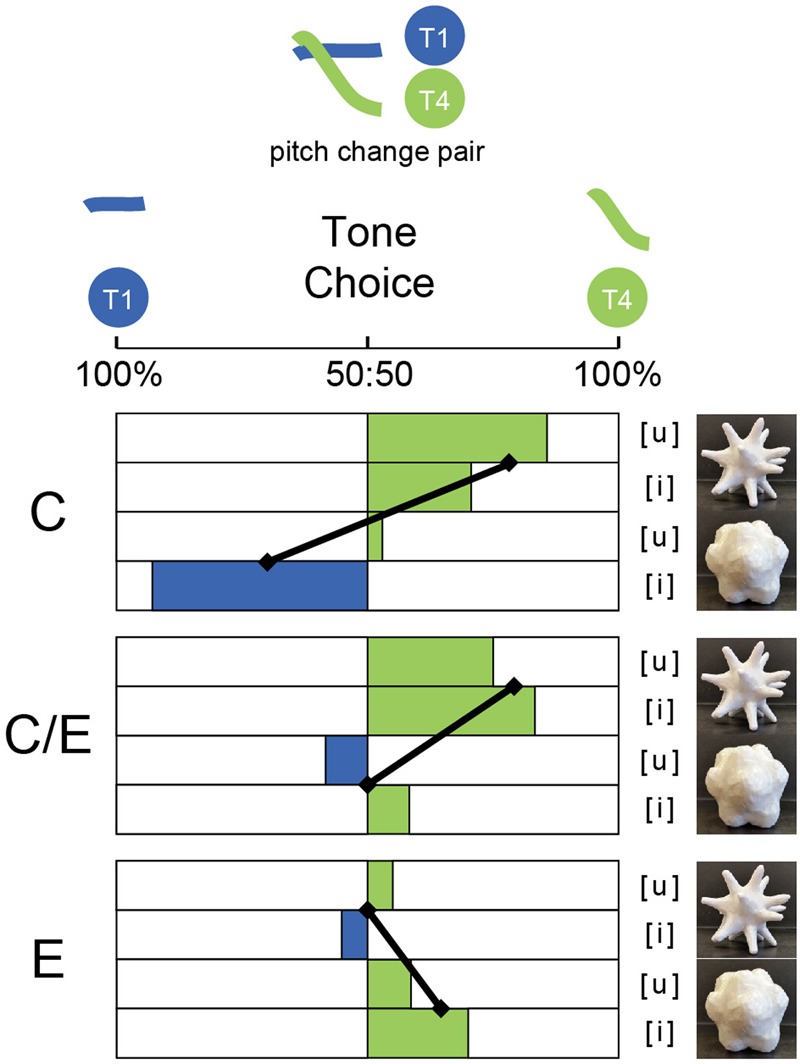
Tone choice for the Tone-1/Tone4 contrast pair in Study 2, where a single visual image was presented with a tone-minimal pair of vowels. Trial types with different visual stimuli (pointy or curvy), and different auditory vowels (/i/, /u/) are shown separately for the three groups, C: Chinese dominant educated in China; Chinese–English balanced bilinguals educated in Singapore; English speakers with no knowledge of Chinese. Angled black lines show group average for each visual stimulus, pooled across vowels (black diamond).

**Figure 6** shows the percentage of participants who made T1 vs. T4 choices for the different shapes. The main effect of shape [*F*(1,202) = 7.829, *p* = 0.006] and the interaction between shape and language [*F*(1,202) = 7.353, *p* = 0.001] were significant. The log odds of choosing Tone 1 for the curvy shape were higher in the Chinese-dominant group (β = 2.559, *p* < 0.001) and in the Chinese-English balanced group (β = 1.919, *p* = 0.015) than in the English group, holding the other effects constant. Overall, regardless of whether the sound was [i] or [u], the difference in the mapping patterns between the shapes and the tones was significant across subject groups. The two Chinese groups (i.e., C and CE) showed a significant ‘T1-curvy, T4-pointy’ pattern, whereas the E group did not. We followed up the language ^∗^ shape interaction with pairwise comparisons between language groups.

In the comparison between the C group and the E group, there was a significant interaction between language and shape [*F*(1,156) = 12.723, *p* < 0.001]. The log odds of choosing Tone 1 for the curvy shape were higher in the Chinese-dominant group (β = 2.559, *p* < 0.001) than in the English group, holding the other effects constant. The results of the Related-Samples McNemar Test demonstrated that the C group showed a significant ‘T1-curvy, T4-pointy’ mapping pattern (*p* = 0.001), whereas the E group did not.

In the comparison between the CE group and the E group, the interaction between language and shape was significant [*F*(1,142) = 6.086, *p* = 0.015]. The log odds of choosing Tone 1 for the curvy shape were higher in the Chinese–English balanced group (β = 1.919, *p* = 0.015) than in the English group, holding the other effects constant. We also conducted pairwise comparisons (Related-Samples McNemar Tests) between shapes for each group. The results demonstrated that like the C group, the CE group also showed a significant ‘T1-curvy, T4-pointy’ mapping pattern (*p* = 0.039), whereas the E group did not (*p* = 0.248).

Lastly, in the pairwise comparison between the two Chinese groups (i.e., C and CE), a significant main effect of shape [*F*(1,106) = 14.042, *p* < 0.001] was found, which did not differ significantly between the two groups. The log odds of choosing Tone 1 for the curvy shape are higher (β = 1.335, *p* = 0.046) than for the pointy shape, holding the other effects constant. Overall, the Chinese speakers shared a ‘T1-curvy, T4-pointy’ tone-shape mapping pattern, which was not observed for the E group.

#### T1/T3 – The Pitch-Height Pair

**FIGURE 7:**
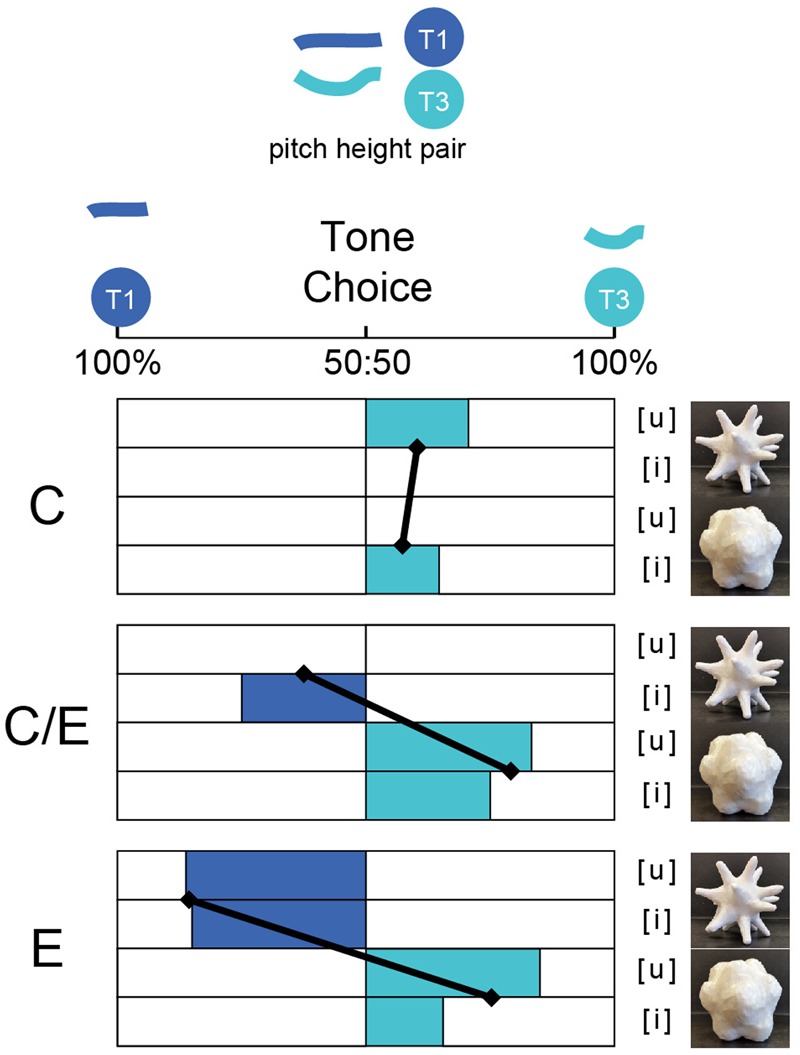
Tone choice for the Tone-1/Tone3 contrast pair in Study 2, where a single visual image was presented with a tone-minimal pair of vowels. Trial types with different visual stimuli (pointy or curvy), and different auditory vowels (/i/, /u/) are shown separately for the three groups, C: Chinese dominant educated in China; Chinese-English balanced bilinguals educated in Singapore; English speakers with no knowledge of Chinese. Angled black lines show group average for each visual stimulus, pooled across vowels (black diamond).

**Figure 7** shows the percentage of participants who made T1 vs. T3 choices for the different shapes. The main effect of shape [*F*(1,202) = 20.469, *p* < 0.001] and the interaction between shape and language [*F*(2,202) = 8.027, *p* < 0.001] were significant. The log odds of choosing Tone 1 for the curvy shape were lower (β = -2.81, *p* < 0.001) than for the pointy shape, holding the other effects constant. The log odds of choosing Tone 1 for the curvy shape were higher in the Chinese-dominant group (β = 2.944, *p* < 0.001) than in the English group, holding the other effects constant. Overall, regardless of whether the sound was [i] or [u], the subject groups showed significantly different mapping patterns between the shapes and the tones. Both the CE group and the E group showed a significant ‘T1-pointy, T3-curvy’ pattern, whereas the C group did not show a clear mapping pattern. To unpack this interaction, further pairwise comparisons were performed.

In the comparison between the C group and the E group, the main effect of Shape [*F*(1,156) = 12.936, *p* < 0.001], the main effect of Language [*F*(1,156) = 4.385, *p* < 0.038], and the interaction between them [*F*(1,156) = 15.659, *p* < 0.001] were all significant. The log odds of choosing Tone 1 for the curvy shape were higher in the Chinese-dominant group (β = 2.944, *p* < 0.001) than in the English group, holding the other effects constant. To unpack this interaction, we conducted pairwise comparisons (Related-Samples McNemar Tests) between shapes for each group. The results demonstrated that the E group showed a significant ‘T1-pointy, T3-curvy’ mapping pattern (*p* < 0.001), on the other hand, the C group did not.

In the comparison between the CE group and the E group, there was only a significant main effect of shape [*F*(1,142) = 30.046, *p* < 0.001] (see Supplementary Table S16). The log odds of choosing Tone 1 for the curvy shape were lower (β = -2.81, *p* < 0.001) than for the pointy shape, holding the other effects constant. Both groups showed a significant ‘T1-pointy, T3-curvy’ mapping pattern between shapes and tones without a significant group difference.

The comparison between the C group and the CE group showed a significant main effect of Shape [*F*(1,106) = 4.04, *p* = 0.047] and a significant interaction between language and shape [*F*(1,106) = 5.405, *p* = 0.022]. The log odds of choosing Tone 1 for the curvy shape were higher in the Chinese-dominant group (β = 1.98, *p* = 0.022) than in the Chinese–English balanced group, holding the other effects constant. Follow-up pairwise comparisons between shapes for each group demonstrated that like the E group, the CE group also showed a significant ‘T1-pointy, T3-curvy’ mapping pattern (*p* = 0.013), which was similar to the E group, but significantly different from the responses of the C group.

### Discussion

Overall, when the vowel was controlled by presenting audio in pairs differing only in tone (tone-minimal pairs), the Chinese-speaking participants’ ‘T1-curvy, T4-pointy’ mapping pattern for the T1-T4 pair was consistent with their mapping pattern for [u] in Study 1 (see **Figures 4, 5**). Previous literature has established that pitch change is important in Chinese speakers’ pitch perception (Jongman et al., 2006; Singh et al., 2014). This is a promising reason why the two Chinese groups matched the more changing Tone 4 with the pointy shape and the less changing T1 with the curvy shape. However, the English-speaking participants did not show the same pattern. Rather, according to the previous literature (Gandour, 1983), English speakers are sensentive to pitch height. As the contrast between T1-T4 is pitch-change-driven (with little contrast in average pitch height), this might help to explain why the English speakers did not show a clear mapping pattern here.

By contrast, for the T1–T3 pair, there was a significant difference in tone-shape mappings across subject groups. Both the English-speaking participants and the Chinese-English balanced participants showed a ‘T1-pointy, T3-curvy’ tone-shape mapping pattern which was consistent with the English-speaking group’s mapping pattern for [u] in Study 1. This is consistent with the previously observed relationships between visual shape and pitch height of pure tones (O’Boyle and Tarte, 1980; Marks, 1987; Walker et al., 2010). In this part of the test, the Chinese-dominant participants did not show a clear mapping pattern. For the Chinese-domiant bilinguals, their weaker English compared to the balanced-bilingual group, and in turn, their stronger sensitity to pitch change might account for the lack of a patterned response to the contrast between T1 and T3.

One curious finding was that the Singaporean Chinese-English bilinguals behaved like the Chinese-dominant group for the T1–T4 pair, but like the English-speaking group for the T1–T3 pair. Although this finding was unexpected, there are some promising explanations for why this may have occurred. First, as the Singaporean education system highlights English as the main language of instructon, the high-low contrast T1–T3 pair might trigger the Chinese–English balanced bilinguals’ English-style pitch-height driven tone perception in the context of a study delivered in English. Since the high/low contrast was made more salient in Study 2, their performance for the English-style mapping pattern shows a perceptual flexibility guided by context – when they hear T1/T4 explicitly contrasted, they behave accoding to the Chinese-dominant steady/dynamic pattern, but when they hear T1/T3 explicitly contrasted, the behave according to the English-speakers’ high/low pattern. Balanced bilinguals’ cross-modal mapping is therefore bi-valent and flexible.

In summary, in terms of the relationship between visual shape and Mandarin Tones, the English-speaking participants showed a ‘high-pointy, low-curvy’ mapping pattern, while the Chinese-dominant participants showed a ‘steady-curvy, dynamic-pointy,’ pattern. That is, the greater the pitch-change within a Mandarin tone, the pointier the shape of its association. The Chinese–English balanced participants, however, seemed to swing between the pitch-height mapping pattern and the pitch-change mapping pattern depending on task types and tone contrasts, showing a kind of perceptual flexibility that has not been previously demonstrated in cross-modal perception or bouba/kiki-type tasks.

## General Discussion

Our online 2AFC tasks replicated the ‘*u*-curvy, *i*-pointy’ vowel-shape cross-modal correspondences, lending support for the universality of this kind of effect for highly contrasting vowels prevalent in the majority of the world’s languages. Confirming the second author’s hypothesis, and consistent with the existing literature on pitch/shape mappings, the English-speaking participants matched visual shape with Mandarin tones according to pitch height, showing a ‘high-pointy, low-curvy’ mapping pattern. Confirming the first author’s native Chinese speaking intuition, the Chinese-dominant participants matched visual shape with Mandarin tones in terms of pitch change showing a ‘steady-curvy, dynamic-pointy’ mapping pattern – that is, the greater the pitch-change within a Mandarin tone, the pointier the shape of its association. Note that these two groups therefore *selected different shapes* for the same stimulus (T1). The Chinese-English balanced bilinguals seemed to swing between the two strategies depending on task types and the salience of the tonal contrasts.

The overwhelming majority of previous research into cross-linguistic, cross-cultural, and developmental studies of sound symbolism has led to a consensus that sound symbolism is essentially universal (e.g., Davis, 1961; Ramachandran and Hubbard, 2001; Maurer et al., 2006; Walker et al., 2010; Kantartzis et al., 2011; Imai and Kita, 2014). Only a small number of published studies challenge this consensus by reporting groups that fail to show the bouba-kiki effect. In Rogers and Ross (1975), a group of Hunjara speakers in Papua New Guinea matched curved and angular line drawings to the test words ‘maluma’ and ‘takete’ essentially at chance. In Styles and Gawne (2017), a group of Syuba speakers in the high foothills of the Himalayas of Nepal matched curved and angular cut-out shapes to test words ‘bubu’ and ‘kiki’ essentially at chance. The linguistic analysis by Styles and Gawne suggested that the differences in cross-modal perception may be due to the match between the test words and the sound structure of the speaker’s languages: They propose that bouba/kiki tests fail if the test words contain sounds or sequences that are unfamiliar to the speaker, and conclude that language-specific perception cascades into cross-modal perception. That is to say, if *sound systems* differ then sound symbolism will necessarily also differ.

In the present study, we observed that Chinese speakers whose language requires more attention to the dynamic aspects of pitch in speech also show crossmodal matching patterns that are aligned with the acoustic feature of pitch change. The low level of pitch variance in Tone 1 aligns with the low degree of visual edge complexity in the curvy shape. By contrast, English speakers show a pattern more in line with previously documented correspondences between high acoustic frequency and high spatial frequency, as the high pitch of Tone 1 aligns with the highly convoluted edges of the pointy shape. This finding therefore aligns with the idea that *differently structured linguistic sound systems generate different patterns of sound-symbolic matching*.

We should note at this stage that it is unclear whether the language-specific differences in pitch-to-shape mapping are the outcome of mapping different tone-features onto a single visual dimension (i.e., English: pitch-height/angularity; Chinese: pitch-change/angularity) or onto different visual features (e.g., English: pitch-height/angularity; Chinese: pitch-change/complexity), since the visual objects used in the study are complex and differ along multiple visual dimensions. While other studies from the same project investigate this question in more detail (Shang, 2017), the current study still provides the first evidence of participants making a completely opposite choice in their match between two stimuli, in a way that can be concretely linked to the attentional properties of speech processing in their language.

To address how linguistic differences could give rise to differences in multisensory processing, it is important to consider the trajectory of language acquisition and neurological specialization for speech. Over the second half of the first year of life, children’s perception for the sounds of speech changes, with gradual ‘tuning’ to the sounds heard in the languages spoken around them (Werker and Tees, 1984; Polka and Bohn, 2003; Kuhl et al., 2006), via a process described as neural commitment (Kuhl, 2004) – a specialized kind of perceptual narrowing. For example, using a headturn task, Kuhl et al. (2006) found that at 6–8 months of age, Japanese infants performed as well as American infants by responding to a change between /ra/ and /la/ with a head-turn, with around 65% accuracy. Two months later, the American infants’ perception had improved substantially, while the Japanese infants dropped to chance on the same task. Since Japanese infants do not typically hear the English sounds [ɺ] and [l] in their native language environment, but American infants do, the change is experience dependent. This model of phonological ‘tuning’ is well known to learners of a foreign language, who often struggle to perceive and produce linguistic elements which were not encoded early in their linguistic experience.

From this perspective, the linguistically tuned adult auditory cortex represents the sounds of speech in a language-specific way, and any pattern of multisensory integration/correspondence would necessarily be biased by the informational properties of that representation. That is to say, if speakers of Chinese have a stronger weighting for the acoustic feature of dynamic pitch change than for the acoustic feature of absolute pitch height, then their multisensory matching will be similarly biased. While the studies reported here did not directly test the auditory perception of the participants, it provides a proof of concept that language-specific perception is evident in the cross-modal perception of speech sounds.

Aside from the statistical regularities provided by language exposure, one alternative has been raised as a possible source of different mapping patterns observed between groups. In a recent study by Chen et al. (2016), Two groups of participants (one from the United States, one from Taiwan) were asked to match “Bouba” or “Kiki” to a number of radial frequency patterns, differing in their frequency (number of bumps) and amplitude (height of bumps), and spikiness (angularity of line composition). The different groups diverged on which of the visual features were dimensionally matched to the auditory stimuli. The authors suggest that well-known East/West perceptual differences may have driven the difference in weighting of visual cues (i.e., holistic versus analytic perceptual processing). While it is hard to make a link between the *particular* visual dimensions in the study and the general literature on holistic versus analytic visual processing (Nisbett and Miyamoto, 2005), it remains a possibility that cultural differences in visual attention play a role in the differences observed in the current study.

To reduce the impact of culturally bound associations, it might be possible to adapt the tone/shape mapping task to methods that remove conscious decision making. Parise and Spence (2012) developed a modified IAT task for testing cross-modal correspondences, where they successfully demonstrated enhanced performance for stimulus combinations including Köhler’s takete/maluma mapping pattern (takete-pointy; maluma-curvy), Sapir’s mil/mal mapping pattern (mil-small; mal-big), along with audiovisual correspondences between auditory pitch and size (high pitch sound and small size; low pitch sound and big size). Methods developed by Hung et al. (2017) include testing linguistic sound-symbolism for written word-forms ‘bubu’ and ‘kiki’ using breaking Continuous Flash Suppression, and audio word forms using masked visual priming. Use of more-automatic methods will add substantially to our understanding of whether the tone-mapping-pattern observed here represents an overt, culturally mediated match, or an automatic outcome of low level perceptual processes.

Finally, it should be noted that the current investigation does not provide any clues into whether one pitch-mapping pattern is the sensory default, and the other, acquired through language-specific tuning processes, whether both are (flexible) defaults but one is lost, or whether neither pattern is predisposed, and both are acquired with exposure. In the process of language acquisition, all three types of ‘tuning’ have been documented for speech sounds, so the same may be true for acoustic properties of pitch (pitch height versus dynamic pitch change). Some hints are provided from the canonical direction of sensory mapping for pitch height in infants (Mondloch and Maurer, 2004), and Chimpanzees (Ludwig et al., 2011) but it should be noted that all of the infants were from non-tone language environments (as indeed were the Chimps), and only non-linguistic pitch stimuli have been tested. It therefore remains unclear whether pitch height, pitch change or some weighted combination of both might be universally implicated prior to language exposure, and which of these features remain as perceptual ‘defaults’ outside the context of language.

## Materials and Data Availability

The audio, and visual stimuli from this project, along with precise task instructions, and the raw data from each participant, are available in an Open Science Framework repository: https://osf.io/364fm.

## Ethics Statement

This study was carried out in accordance with the recommendations of IRB of Nanyang Technological University with online informed consent from all subjects. All subjects gave online informed consent in accordance with the Declaration of Helsinki. The protocol was approved by the IRB of Nanyang Technological University.

## Author Contributions

NS and SJS conceived of the study. NS collected the data. NS and SJS analyzed the data. NS and SJS wrote the paper. SJS prepared the final version of the figures.

## Conflict of Interest Statement

The authors declare that the research was conducted in the absence of any commercial or financial relationships that could be construed as a potential conflict of interest.

## Footnotes

1 https://osf.io/364fm
