# Supplementary material for: Is a High Tone Pointy? Speakers of Different Languages Match Mandarin Chinese Tones to Visual Shapes Differently
Source: Front Psychol. 2017 Dec 7;8:2139. doi: 10.3389/fpsyg.2017.02139 (PMC5726031; doi:10.3389/fpsyg.2017.02139)
Supplement: Supplementary file 1 [file Data_Sheet_1.pdf]

Supplementary Materials

Shang N and Styles SJ (2017) Is a High Tone Pointy? Speakers of Different Languages Match Mandarin Chinese Tones to Visual Shapes Differently. *Front. Psychol.* 8:2139. doi: 10.3389/fpsyg.2017.02139

Table S1. Self-proficiency rating scale

1.	"I can speak easily about most things without thinking about how to say them"
2.	"I can talk about many things, but I often need to think about how to put my words together when I speak"
3.	"I can understand most of what people are saying but I can only think how to reply sometimes"
4.	"I can understand some of what people are saying but I usually can't think how to reply"
5.	"I have taken lessons and have begun practicing how to say certain kinds of words and sentences"

Table S2. Self-reported language levels

Group	Language Level (Range)	
	Chinese	English
C	1	2-4
C/E	1-2	1-3
E	N/A	1-2

Table S3. Rating of typicality of the auditory stimuli

Sounds	Median	Range
i1	6	5-7
i2	6	3-7
i3	6	4-7
i4	5	4-7
u1	5	4-7
u2	5	3-7
u3	6	4-7
u4	5	3-7

Full Statistical reports

Study 1: Two shapes with one sound

Table S4. GLMM Fixed Effects (Study 1, overall)

	F	df1	df2	Sig.
Vowel	140.108	1	776	.000
Tone	4.538	3	776	.004
Language	1.184	2	776	.307
Language * Vowel	1.869	2	776	.155
Language * Tone	1.867	6	776	.084
Vowel * Tone	.959	3	776	.411
Language * Vowel * Tone	1.09	6	776	.367

Table S5. GLMM Fixed Effects (Study 1, [i])

	F	df1	df2	Sig.
Tone	4.052	3	388	.007
Language	1.19	2	388	.305
Language * Tone	1.71	6	388	.117

Table S6. GLMM Fixed Effects (Study 1, [u])

	F	df1	df2	Sig.
Tone	4.807	3	388	.003
Language	.946	2	388	.393
Language * Tone	2.82	6	388	.011

Table S7. GLMM Fixed Effects (Study 1, [u], CE : ENG)

	F	df1	df2	Sig.
Tone	1.957	3	212	.121
Language	.177	1	212	.674
Language * Tone	3.013	3	212	.031

Table S8. Pairwise comparisons between tones (Study 1)

		C/E	C	E
Vowel	Tone comparison	*p*	*p*	*p*
u	1&2	.180	.013	.332
u	1&3	.109	.049	.302
u	1&4	.007	.003	.607
u	2&3	1.000	.503	>.999
u	2&4	.180	1.000	.754
u	3&4	.302	.359	.754
u	1&4 vs. 2&3	>.999	243	.041

Table S9. GLMM Fixed Effects (Study 1, [u], C: ENG)

	F	df1	df2	Sig.
Tone	1.63	3	272	.183
Language	.754	1	272	.386
Language * Tone	3.784	3	272	.011

Table S10. GLMM Fixed Effects (Study 1, [u], C: CE)

	F	df1	df2	Sig.
Tone	12.279	3	292	<.001
Language	1..574	1	292	.211
Language * Tone	.099	3	292	.961

Study 2: Two sounds with one shape

T1/T4 – The Pitch-Change Pair

Table S11. GLMM Fixed Effects (Study 2, T1-T4, overall)

	F	df1	df2	Sig.
Shape	7.829	1	202	.006
Language	.639	2	202	.529
Language * Shape	7.353	2	202	.001

Table S12. GLMM Fixed Effects (Study 2, T1-T4, CE vs. E)

	F	df1	df2	Sig.
Shape	.931	1	142	.336
Language	1.144	1	142	.287
Language * Shape	6.086	1	142	.015

Table S13. GLMM Fixed Effects (Study 2, T1-T4, C vs. E)

	F	df1	df2	Sig.
Shape	3.753	1	156	.055
Language	<.001	1	156	.986
Language * Shape	12.723	1	156	<.001

Table S14. GLMM Fixed Effects (Study 2, T1-T4, C vs. CE)

	F	df1	df2	Sig.
Shape	14.042	1	106	<.001
Language	<.001	1	106	.341
Language * Shape	12.723	1	106	.471

T1/T3 – The Pitch-Height Pair

Table S15. GLMM Fixed Effects (Study 2, T1-T3, overall)

	F	df1	df2	Sig.
Shape	20.469	1	202	<.001
Language	2.753	2	202	.066
Language * Shape	8.027	2	202	<.001

Table S16. GLMM Fixed Effects (Study 2, T1-T3, CE vs. E)

	F	df1	df2	Sig.
Shape	30.046	1	142	<.001
Language	3.536	1	142	.062
Language * Shape	1.289	1	142	.258

Table S17. GLMM Fixed Effects (Study 2, T1-T3, C vs. E)

	F	df1	df2	Sig.
Shape	12.936	1	156	<.001
Language	4.385	1	156	.038
Language * Shape	15.659	1	156	<.001

Table S18. GLMM Fixed Effects (Study 2, T1-T3, C vs. CE)

	F	df1	df2	Sig.
Shape	4.04	1	106	.047
Language	.002	1	106	.963
Language * Shape	5.405	1	106	.022

Supplementary Figures

Study 1: Two shapes with one sound

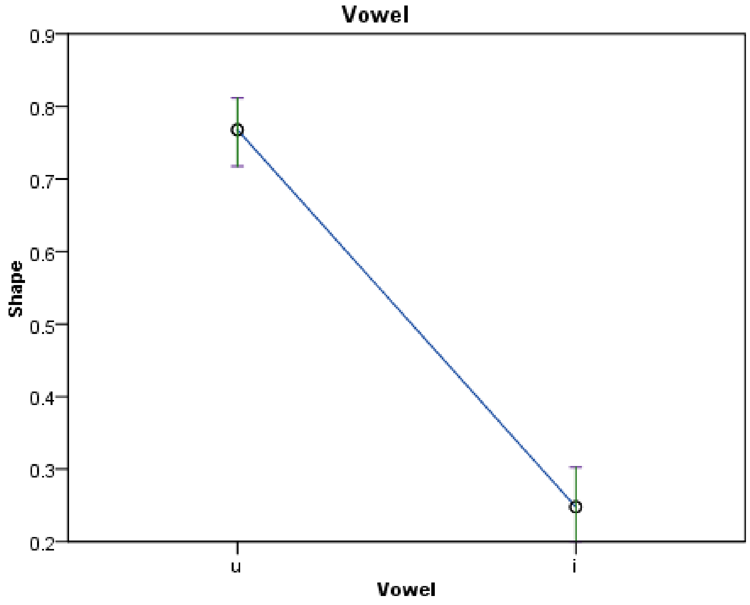

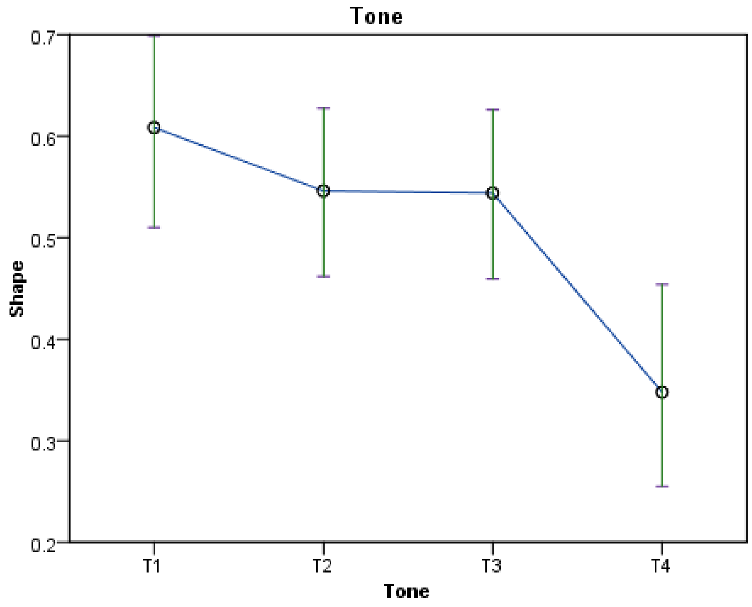

Figure S1. Estimated Means for significant effects (0=pointy) (Study 1: overall)

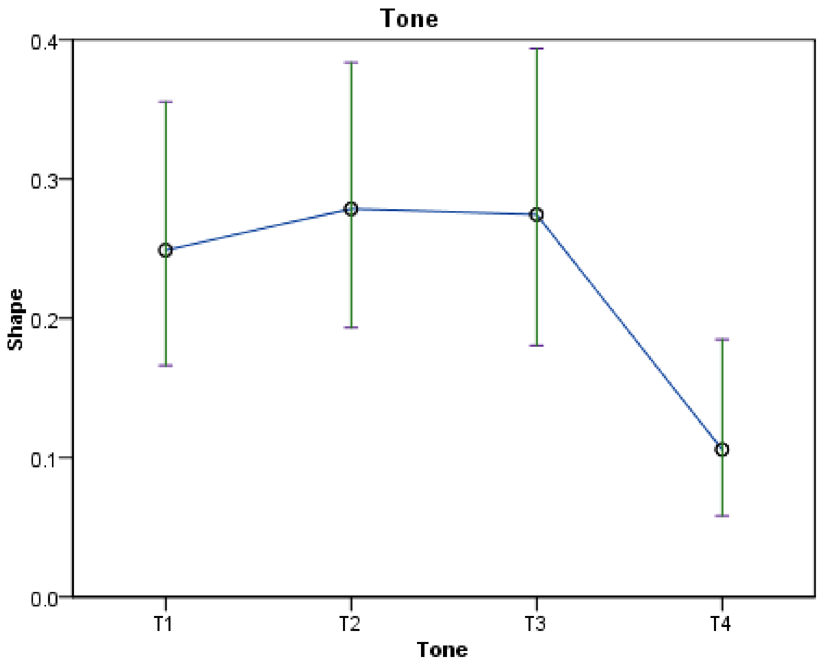

Figure S2. Estimated Means for significant effects (0=pointy) (Study 1: [i])

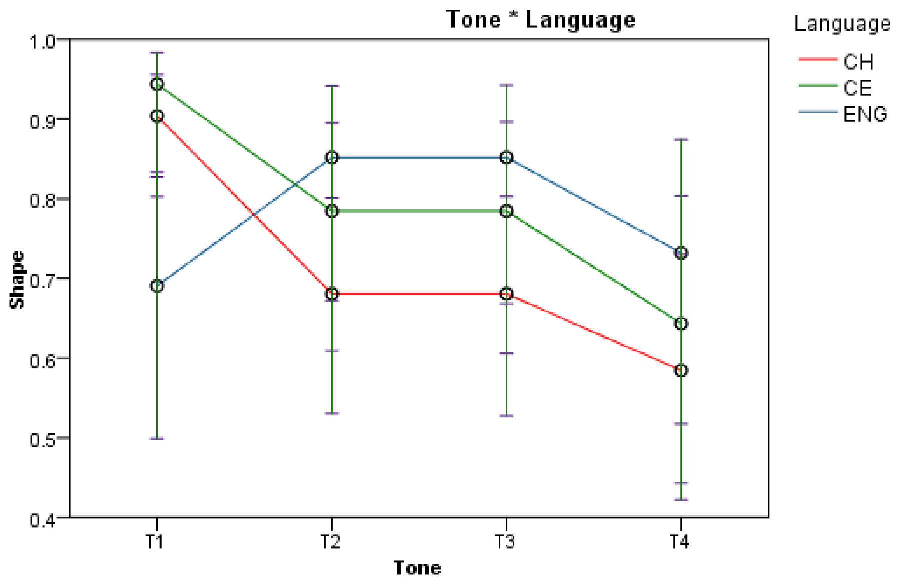

Figure S3. Estimated Means for significant effects (0=pointy) (Study 1: [u])

Study 2: Two sounds with one shape

T1/T4 – The Pitch-Change Pair

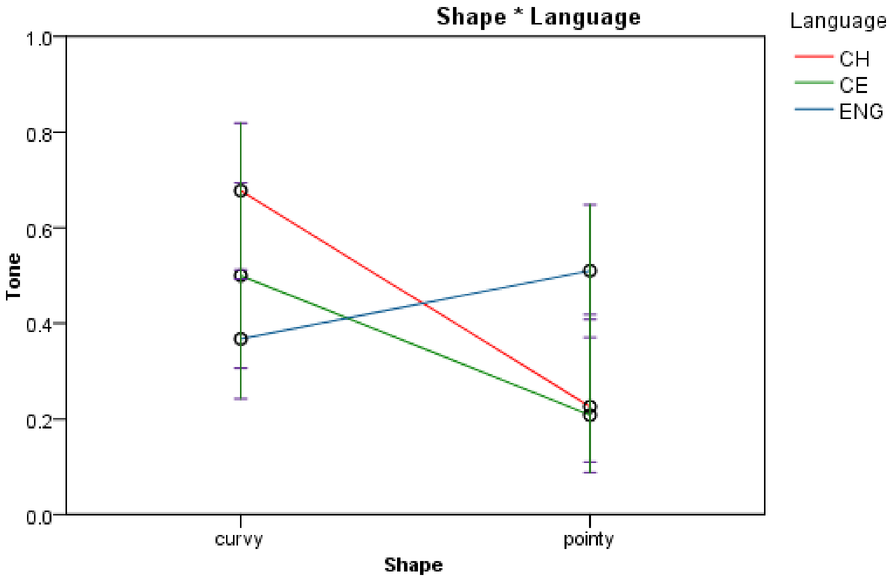

Figure S4. Estimated Means for significant effects (0=T4) (Study 2, T1-T4: overall)

T1/T3 – The Pitch-Height Pair

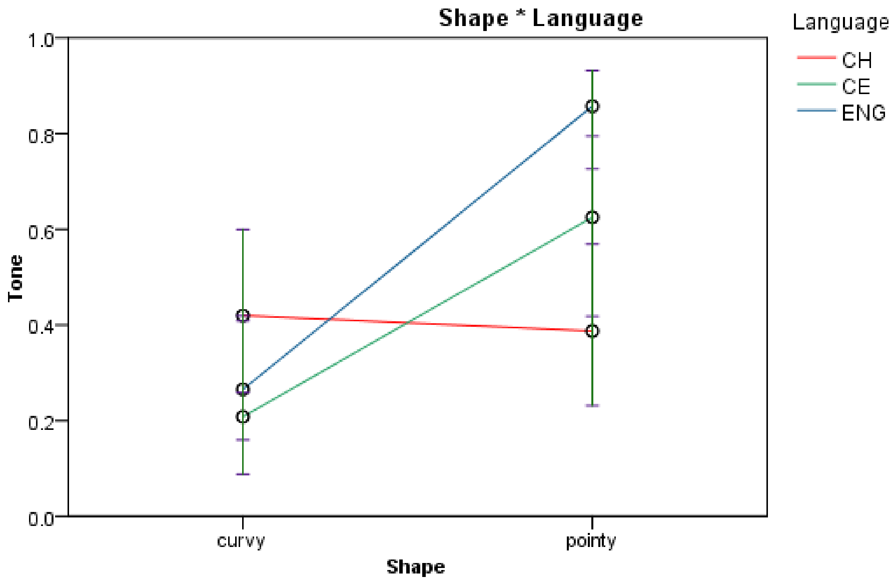

Figure S5. Estimated Means for significant effects (0=T3) (Study 2, T1-T3: overall)
